# DTCNet: finger flexion decoding with three-dimensional ECoG data

**DOI:** 10.3389/fncom.2025.1627819

**Published:** 2025-07-09

**Authors:** Fufeng Wang, Zihe Luo, Wei Lv, XiaoLin Zhu

**Affiliations:** School of Big Data, Zhuhai College of Science and Technology, Zhuhai, China

**Keywords:** ECoG signals, brain-computer interfaces, finger movement trajectories, 3D spatio-temporal spectrograms, dilated-transposed convolution

## Abstract

ECoG signals are widely used in Brain-Computer Interfaces (BCIs) due to their high spatial resolution and superior signal quality, particularly in the field of neural control. ECoG enables more accurate decoding of brain activity compared to traditional EEG. By obtaining cortical ECoG signals directly from the cerebral cortex, complex motor commands, such as finger movement trajectories, can be decoded more efficiently. However, existing studies still face significant challenges in accurately decoding finger movement trajectories. Specifically, current models tend to confuse the movement information of different fingers and fail to fully exploit the dependencies within time series when predicting long sequences, resulting in limited decoding performance. To address these challenges, this paper proposes a novel decoding method that transforms 2D ECoG data samples into 3D spatio-temporal spectrograms with time-stamped features via wavelet transform. The method further enables accurate decoding of finger bending by using a 1D convolutional network composed of Dilated-Transposed convolution, which together extract channel band features and temporal variations in tandem. The proposed method achieved the best performance among three subjects in BCI Competition IV. Compared with existing studies, our method made the correlation coefficient between the predicted multi-finger motion trajectory and the actual multi-finger motion trajectory exceed 80% for the first time, with the highest correlation coefficient reaching 82%. This approach provides new insights and solutions for high-precision decoding of brain-machine signals, particularly in precise command control tasks, and advances the application of BCI systems in real-world neuroprosthetic control.

## 1 Introduction

The electrocorticogram (ECoG) is a brain signal recording method known for its high spatial resolution and superior signal quality, offering significant advantages in brain-computer interfaces (BCIs) and motion control. By directly placing electrodes on the cortical surface, ECoG can precisely capture neural oscillations associated with movement, such as μ-rhythm, β waves, and γ waves (Ball et al., 2008; Marjaninejad et al., 2017; Tam et al., 2019). Compared to traditional Electroencephalography (EEG), ECoG greatly enhances spatial resolution, overcoming the attenuation and diffusion of electrical signals caused by the skull and scalp, thereby enabling more accurate localization of specific brain activity. Additionally, ECoG provides superior signal stability and noise suppression compared to EEG, effectively reducing interference from artifacts like electromyographic (EMG) signals and eye movements, thus improving the accuracy of limb motion decoding (Schalk and Leuthardt, 2011; Toro et al., 1994). Particularly in tasks that require high precision and low latency, such as finger trajectory decoding, ECoG offers more reliable and timely signal feedback (Hill et al., 2006; Leuthardt et al., 2004; Shenoy et al., 2008).

In recent years, numerous studies have explored the motion control applications of ECoG signals, particularly in the context of decoding ECoG signals using machine learning or deep learning models. Some studies focus on classifying finger activations (Onaran et al., 2011; Saa et al., 2016; Shenoy et al., 2007), while others target the decoding of finger bending trajectories (Volkova et al., 2019). Flamary and Rakotomamonjy (2011) and Liang and Bougrain (2012) sought to decode finger flexion using the publicly available BCI Competition IV dataset (Schalk et al., 2007)). Flamary proposed a switching linear regression method, while Liang used a frequency-band-specific ECoG amplitude modulation linear regression method. Both approaches secured first and second place in the competition, respectively. With the continuous advancement of deep learning technologies, methods like Convolutional Neural Networks (CNNs) (LeCun and Bengio, 1995) and Recurrent Neural Networks (RNNs) (Hochreiter and Schmidhuber, 1997) have been widely applied to ECoG decoding tasks (Ingolfsson et al., 2020; Lawhern et al., 2018; Song et al., 2023). For instance, Xie et al. (2018) used a CNN-LSTM (Shi et al., 2015) architecture to decode finger trajectories; Frey et al. (2021) developed a 2D convolutional decoder for ECoG finger trajectory regression; Petrosyan et al. (2021) proposed a compact and interpretable CNN architecture that allowed for biologically interpretable spatial and temporal patterns; Yao et al. (2022) introduced a new feature based on Riemannian geometry for finger motion decoding and employed the LightGBM (Ke et al., 2017) model, significantly improving continuous finger trajectory decoding while reducing training and inference times; and Lomtev et al. (2023) used a convolutional encoding-decoding architecture with skip connections to further enhance motion trajectory decoding performance. Recent advances in 2024 have further demonstrated the versatility of CNNs architectures across biomedical signal processing domains. Attention mechanisms have been successfully integrated with CNNs for enhanced feature extraction in neuroimaging applications (Rasheed et al., 2024b), while hybrid CNN approaches have shown promising results in multi-modal brain signal classification tasks (Ahmed et al., 2024; Rasheed et al., 2024a). These developments highlight the growing trend toward multi-feature fusion architectures that combine convolutional layers with ensemble methods for improved signal decoding performance (Ahmad et al., 2024). Such innovations in neural network design provide valuable insights for advancing ECoG based motion control systems, particularly in terms of feature representation learning.

Traditional regression methods (Flamary et al., Liang et al.) provide computational efficiency but are limited by linear assumptions that cannot capture the nonlinear neural-motor dynamics. CNN-based approaches (Xie et al., Frey et al.) excel at spatial feature extraction but struggle with long-range temporal dependencies crucial for continuous motion decoding. CNN-LSTM hybrids address temporal modeling but suffer from vanishing gradients and sequential processing limitations. Recent encoder-decoder architectures (Lomtev et al.) preserve detailed information through skip connections but remain constrained by standard convolutions’ limited receptive fields for multi-scale temporal pattern modeling. To address these challenges, we propose a novel decoding method based on prior research. Specifically, we constructed 3D ECoG data samples by calculating spectrograms using wavelet transforms (Hazarika et al., 1997) and employed a dilated transpose convolutional network to decode finger bending. The dilated convolution captures temporal dependencies between electrode and frequency signals while improving computational efficiency, and the transpose convolution restores temporal resolution, optimizing the decoding process. Our model shows a significant improvement in performance over previous approaches. The main contributions of this study can be summarized as follows:

Overlapping Sliding Window Technique: We segment long time-series data with multi-channel frequency-band information into smaller time windows, increasing the diversity of training samples while preserving information from both previous and subsequent time points. This approach helps the model better understand long-term dependencies and local variations in the signal.

• Feature Extraction Stage: We use 1D dilated convolutions, which not only enhance the interaction between signals from different electrodes and frequency bands but also capture their temporal dependencies. Compared to traditional 2D convolution methods, dilated convolutions reduce the dimensionality of the input features by merging the electrode and frequency dimensions into a unified feature space, improving computational efficiency and reducing model complexity.
• Decoding Stage: We use transposed convolutions to restore temporal resolution and integrate low-level and high-level features via skip connections. This allows the model to generate appropriate interpolation patterns from low-resolution feature maps, thereby recovering high-frequency details and improving the numerical precision of the signal.

## 2 Materials and methods

### 2.1 Dataset description

**FIGURE 1:**
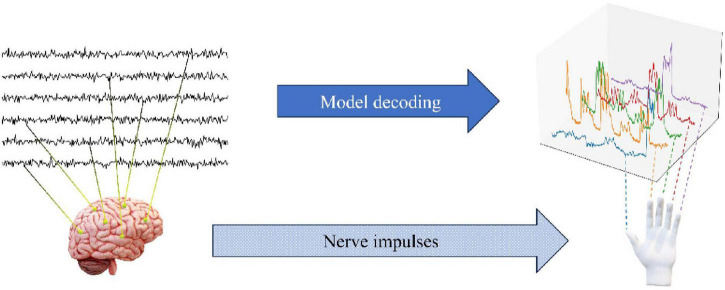
ECoG decoding finger flexing overview view.

The dataset employed in this study is derived from the publicly accessible BCI Competition IV dataset and comprises electrocorticography (ECoG) signal recordings from three subjects. The ECoG signals for each subject were acquired using the BCI2000 system. Subsequently, the signals were subjected to a band-pass filtering process between 0.15 and 200 Hz, with a recorded sampling rate of 1,000 Hz. It is important to note that the order of the electrode channels has been disrupted by the data provider, which has resulted in a lack of information regarding the spatial distribution of the electrodes. In the experimental task, the subjects were required to perform finger movements in accordance with word commands displayed on a computer screen. Each cue lasted for 2 s, followed by a 2-s rest period during which the screen was blank. During each cue, subjects typically repeated the movement of the cued finger three to five times, and the bending angle of the finger was recorded at 25 Hz using a data glove. The experiment lasted for 10 min per subject. To create the training and test sets, the tournament organizers divided the 10-min ECoG signal chronologically into a 6 min 40 s training set 400k samples and a 3 min 20 s test set 200 k samples. Figure 1 shows how to decode the degree of finger bending from the ECoG signal collected by the BCI system.

### 2.2 Dataset preprocessing

In the data preprocessing stage, this study follows the method described by Lomtev et al. (2023). The ECoG signals, initially recorded at 1,000 Hz, and the finger flexion data, recorded at 25 Hz, were both resampled to a common rate of 100 Hz. The primary aim of this preprocessing is twofold: first, to preserve the temporal characteristics of the signals, ensuring the integrity of time-dependent information; and second, to reduce the data volume by lowering the temporal resolution, thereby improving the efficiency of model training.

#### 2.2.1 FingerFlexion data preprocessing

In the preprocessing of labeled data, the scaling ratio between the ECoG signals and the finger bending data sampling rate is first calculated. The finger bending data is then interpolated using cubic interpolation to increase its sampling rate from 25 to 100 Hz. This process ensures temporal alignment between the finger bending data and the ECoG signals, enabling the construction of 3D signal samples.

#### 2.2.2 ECoG data preprocessing

**FIGURE 2:**
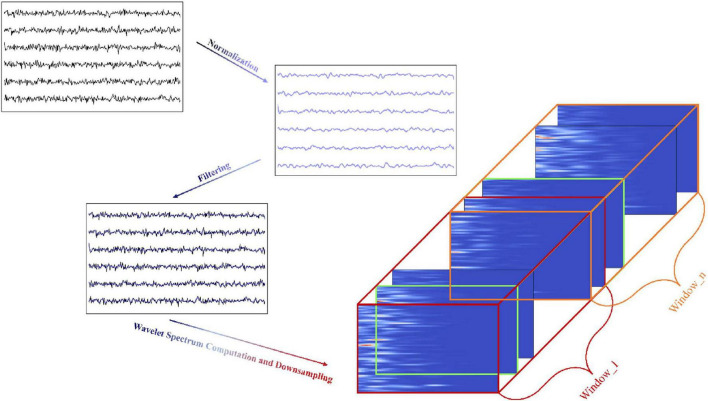
Flowchart of data preprocessing and sample construction.

Figure 2 shows the data preprocessing process from raw data through normalization, filtering, wavelet spectrum calculation, and finally constructing 3D samples by time window segmentation.

Normalization. To eliminate amplitude differences between channels and ensure consistent magnitude and distribution across all channels, the mean and standard deviation of each channel are first calculated, followed by normalization of the signals. To more accurately reflect the activity of different brain regions, the median of each channel is then removed from the normalized signals.

Filtering. In the next step, the ECoG signals are processed using a bandpass filter with a frequency range of 40–300 Hz to remove physiological noise below 40 Hz and high-frequency artifacts above 300 Hz. A notch filter is then applied to remove the 60 Hz power line frequency and its harmonics, further reducing power line interference.

Wavelet Spectrum Computation and Downsampling. In the final step, the filtered ECoG signal is downsampled using the Morlet wavelet transform to generate a spectrogram. This is achieved by applying a set of frequencies, uniformly distributed on a logarithmic scale (ranging from 40 to 300 Hz), with the aim of capturing the time localized features of the different frequency components.

The spectrogram produced by the Morlet wavelet transform is represented as a three-dimensional matrix: electrode channels, wavelet frequencies, and time. Each channel in the spectrogram displays the power distribution of the signal across distinct frequency bands. To ensure alignment with the labeled samples at the same sampling rate, the time dimension was downsampled from the original 1 kHz to 100 Hz, maintaining consistency with the subsequent model input.

The wavelet transform applied here converts a signal with an input shape of (electrode_channel, time) into a spectrogram with an output shape of (electrode_channel, wavelet_frequency, time), thereby revealing the time-frequency characteristics of the ECoG signal through time-frequency analysis. The Morlet wavelet function is expressed as follows:


(1) $$\psi_{f}\,\left(t\right)\,A\cdot e^{-\frac{t^{2}}{2\,\sigma^{2}}}\cdot e^{2\,\pi \,i\,f\,t}$$

(2) $$\sigma =\frac{n_{c\,y\,c\,l\,e\,s}}{2\,\pi \,f}$$

where *A* is a normalization constant, σ controls the width of the Gaussian envelope, $e^{-{\left(t/\sqrt{2}\,\sigma \right)}^{2}}$ represents the Gaussian envelope function that adjusts the temporal window width of the wavelet, and *n*_*cycles*_ determines the number of cycles that balance time and frequency resolution, while *f* determines the wavelet’s center frequency of the sinusoidal part. In the core step of the wavelet transform, for each electrode channel *c* and each frequency *f* the wavelet coefficients *W*_*c,f,t*_ are computed using the following equation:


(3) $$W_{c,f,t}=X_{c}*\psi_{f}\,\left(t\right)$$

where * denotes the convolution operation, *X_c_* is the number of electrode channels in the input signal, and ψ_*f*_ is the Morlet wavelet function. After this calculation, the signal for each electrode channel is convolved with the wavelet function of the corresponding frequency, and the wavelet coefficient at time *t*, electrode channel *c* and frequency *f* is obtained. The final output of the wavelet transform has a shape of (*c*, *f*, *t*), where *c* refers to the number of electrode channels, *f* to the number of wavelet frequencies, and *t* to the number of time samples.

#### 2.2.3 Construction of the 3D dataset

Using a sliding time window with a step size of 1 and a window length of 256 (adapted to the transpose-convolution operation in the subsequent model), the ECoG data with shape (*c*, *f*, *t*) is segmented. Simultaneously, the finger bending data with shape of (finger, *t*) is matched based on the same time points, and the total *T* time points are reassembled into *N*_*samples*_ new data samples. After segmentation, each data sample has the shape (*c*, *f*, *l*), where the *l* = 256 represents the number of time samples within the window. The number of samples, *N*_*samples*_ generated by the sliding window is calculated as:


(4) $$N_{s\,a\,m\,p\,l\,e\,s}=\left\lfloor \frac{T-l}{s}\right\rfloor +1$$

This sliding window approach divides the original long time series into multiple fixed-length samples. The step size *s* of the sliding window determines the degree of overlap between samples, while the window size *l* defines the time span of each sample. where *s* = 1 represents the step size of the sliding window.

### 2.3 Model architecture

#### 2.3.1 Backbone network

**FIGURE 3:**
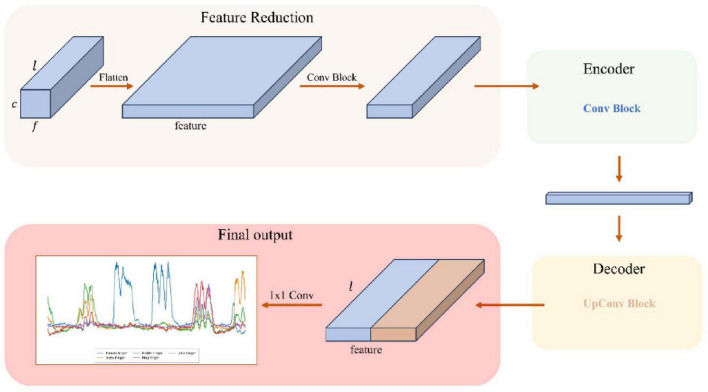
General framework of the model.

Figure 3 shows the overall framework of the model, including feature dimension reduction, encoder feature extraction, decoder feature reconstruction, and final convolution output.

**FIGURE 4:**
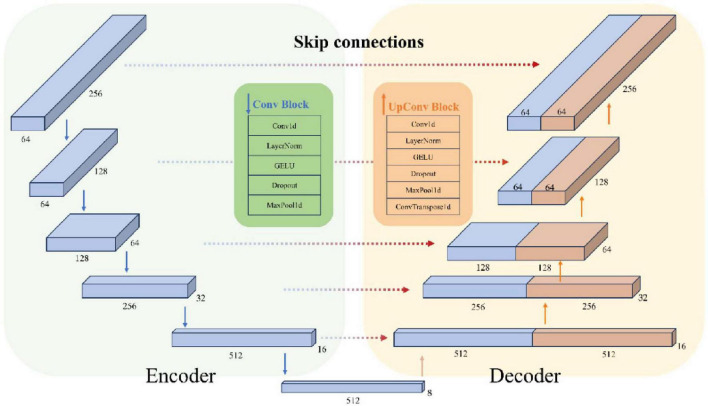
Structure of encoder module and decoder module.

Figure 4 shows the structure of the encoder and decoder modules in detail, including feature extraction and downsampling through convolution blocks, and feature reconstruction and upsampling through upconvolution blocks and skip connections. The Feature Reduction module processes the raw input data by reshaping and applying convolution, followed by the Encoder, which compresses these features to capture essential temporal, frequency, and spatial information. Next, the Decoder reconstructs the temporal resolution using transposed convolution and integrates low-level and high-level features through skip connections. This process allows the model to recover detailed temporal information and ultimately predict the bending values of the five fingers. The model parameter count varies by subject (550–790k parameters) as the Feature Reduction layer adapts to different electrode sampling configurations across subjects.

#### 2.3.2 Feature Reduction

The original input data has a shape of (*c*, *f*, *l*), which is first reshaped into (batchsize, feature, *l*), where the feature dimension represents the combined features of all electrodes and frequency bands at each time point. The purpose of the 1D dilated convolution in the feature dimension is to extract information across multiple electrode channels and frequency bands through the convolution operation.

#### 2.3.3 Encoder

The task of the Encoder layer is to extract and compress the temporal, frequency, and spatial features of the input data, ultimately obtaining a multiscale high-dimensional representation.

First, 1D dilated convolution is applied to the feature dimensions to capture the temporal representation of each feature dimension. Next, layer normalization, GELU activation, and a Dropout layer are applied sequentially. Finally, the temporal dimension is downsampled using a max pooling layer to reduce the temporal resolution. After the downsampling step of each encoder layer, a pooled copy is saved for model skip connections. After five encoder layer downsampling processes, a final high-dimensional representation is obtained.

#### 2.3.4 Decoder

The Decoder layer consists of a convolutional module and a transposed convolutional module, designed to reconstruct the temporal resolution by incorporating skip connections.

Skip concatenation combines the low-level detail information obtained during the downsampling phase with the high-level abstract features. The concatenated feature map doubles the size of the feature in its second dimension, which is then processed by a convolution module using conventional 1D convolution. Subsequently, the timing information, which was compressed into the deep convolutional kernel receptive fields during downsampling, is reconstructed by applying a transposed convolution to recover the temporal resolution.

Finally, the features obtained from the decoder are mapped to five finger bend values using 1x1 convolutional layer. This convolutional kernel performs a weighted summation across channels at each position and each time point, and the output at each time step is represented as a vector of length 5, with each component corresponding to a finger bend value.

## 3 Results

### 3.1 Experimental details

The experiment was conducted on a personal computer equipped with an Intel i7–14700KF CPU, 96 GB of RAM, and an NVIDIA 4070TI SUPER GPU with CUDA acceleration. The model was implemented using Python 3.11.7 and the PyTorch Lightning framework. The computational environment was configured within a Docker container based on the nvcr.io/nvidia/pytorch:24.10-py3 image, which includes PyTorch 2.5.0, CUDA 12.6, and cuDNN 9.5.0, ensuring optimal efficiency and compatibility.

In this experiment, the Adam optimiser was used for model training, the learning rate was set to 8.42e-5, with reference to Lomtev et al. (2023), and L2 regularization technique was introduced to prevent overfitting, and the weight decay coefficient was set to 1e-6. The encoder adopts a multilayer feature extraction architecture, with the feature dimensions of each layer being, in order, (64, 64, 128, 256, 512, 512). Among them, the first 64-dimensional feature extraction layer serves as a feature dimensionality reduction module and uses 3 × 3 convolution kernel for standard convolution operation. The convolution kernel sizes of the subsequent layers are set to (7, 7, 5, 5, 5), and the corresponding dilation convolution expansion rates are (1, 2, 3, 1, 2), forming a sawtooth expansion pattern to enhance the sensory field coverage. To prevent overfitting, the dropout rate was uniformly set to 0.1. To ensure the model generalization performance, this study constructed an independent training model for each subject, but the hyperparameter tuning process was completed on a single subject’s data only, and the optimal hyperparameter configurations were subsequently applied to all subjects. This strategy effectively avoids overfitting the model to specific subjects and ensures the generalisability of the performance improvement. The loss function combines mean squared error (MSE) and cosine similarity to optimize model performance in terms of both numerical accuracy and trend prediction. Specifically, the mean squared error loss is used to assess the numerical error in the model’s prediction of finger bending angles, while cosine similarity evaluates the alignment between the predicted values and the direction of change in the true bending angles. The cosine similarity is calculated as shown in Equation (5):


(5) $$C\,o\,s\,i\,n\,e\,S\,i\,m\,i\,l\,a\,r\,i\,t\,y\,\left(x_{i},y_{i}\right)=\frac{x_{i}\cdot y_{i}}{||x_{i}||\,||y_{i}||}$$

Where *x*_*i*_⋅*y*_*i*_ denotes the dot product of the vectors *x_i_* and *y_i_*, with *x_i_* and *y_i_* representing the *i*-th sample in the vectors *x* and *y*, respectively, and ||*x*_*i*_|| and ||*y*_*i*_|| denote the Euclidean norms of *x_i_* and *y_i_*.

### 3.2 Evaluation metrics

We used the Pearson correlation coefficient as the key metric for model evaluation, which was calculated according to the criteria provided by the organizers of the BCI competition. The Pearson correlation coefficient is used to quantify the linear relationship between the predicted value and the true value, and is calculated using the following formula:


(6) $$r=\frac{\Sigma_{i=1}^{n}\,\left(x_{i}-\bar{x}\right)\,\left(y_{i}-\bar{y}\right)}{\sqrt{\sum_{i=1}^{n}{\left(x_{i}-\bar{x}\right)}^{2}\,\sum_{i=1}^{n}{\left(y_{i}-\bar{y}\right)}^{2}}}$$

The specific calculation process involves first computing the means x^–^ and y^–^ of the predicted values x_i and the true values y_i, respectively. Next, the deviations of each predicted and true value from their respective means are calculated. The product of these deviations is then summed to obtain the covariance, which is subsequently normalized using the standard deviations of the predicted and true values to compute the Pearson correlation coefficient.

### 3.3 Comparison study

Tables 1, 2 present a comparative analysis of various methods and the performance of our proposed solution on the BCI Competition IV Dataset 4. Table 1 summarizes the performance of several approaches, evaluated across three subjects (S1, S2, and S3), and reports the average accuracy for each subject. The methods range from traditional machine learning models to more recent deep learning architectures. Table 2 provides a more detailed analysis of the performance of our model on individual fingers for each subject. The average accuracy across all fingers (Thumb, Index, Middle, Ring, and Little) is 0.69, with the highest performance observed on S3, where accuracies range from 0.72 (Middle) to 0.82 (Thumb) for each finger.

**TABLE 1:** Performance comparison on the BCI competition IV dataset 4 for different methods.

Method	S1	S2	S3	Average
Switching linear models (2012)	0.48	0.24	0.56	0.43
Interpretable Compact CNN (2021)	0.45	0.34	0.56	0.45
Linear regression based on band-specific ECoG (2012)	0.45	0.39	0.59	0.48
CNN-LSTM (2018)	0.56	0.41	0.58	0.52
Multi purpose CNN (2021)	N/A	N/A	N/A	0.52
lightGBM (2022)	0.52	0.47	0.61	0.53
FingerFlex (2023)	0.64	0.56	0.73	0.64
Our solution	0.71	0.59	0.77	0.69

**TABLE 2:** Performance of our model on each finger for each subject.

Subject	Thumb	Index	Middle	Ring	Little	Avg
S1	0.69	0.76	0.76	0.74	0.60	0.71
S2	0.62	0.67	0.52	0.56	0.58	0.59
S3	0.82	0.73	0.72	0.81	0.77	0.77
Average	0.70	0.72	0.67	0.70	0.65	0.69

S3 demonstrates strong overall performance, with the Thumb and Ring fingers achieving accuracies exceeding 0.80. In contrast, S2 exhibits relatively lower performance across all models, particularly for the Middle finger (0.52). This lower performance in S2 can be attributed to the participant having fewer effective recording channels, resulting in greater instability during the decoding process.

**FIGURE 5:**
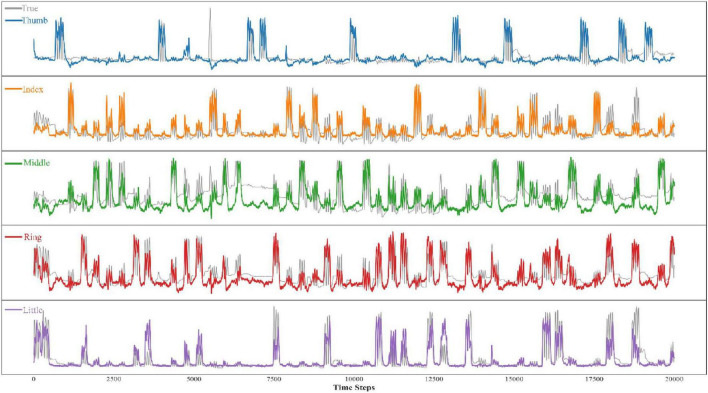
Visualization of predictions for five fingers of a subject.

The results from both tables highlight the robustness and adaptability of our model across various subjects and finger-specific tasks. Compared to existing methods, our solution demonstrates superior performance, particularly for subjects with more complex data patterns, confirming its potential for practical applications in Brain-Computer Interface (BCI) systems. Figure 5 shows the comparison between the predicted and actual results of bending the five fingers of one of the subjects.

**FIGURE 6:**
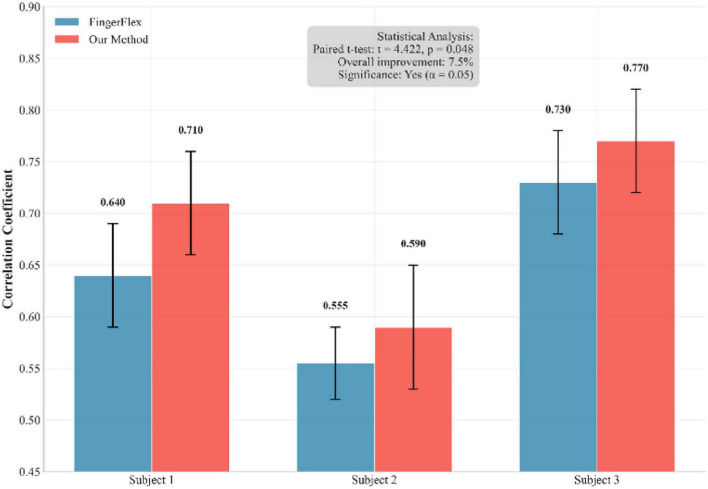
Performance comparison between our method and FingerFlex across multiple independent training runs.

**FIGURE 7:**
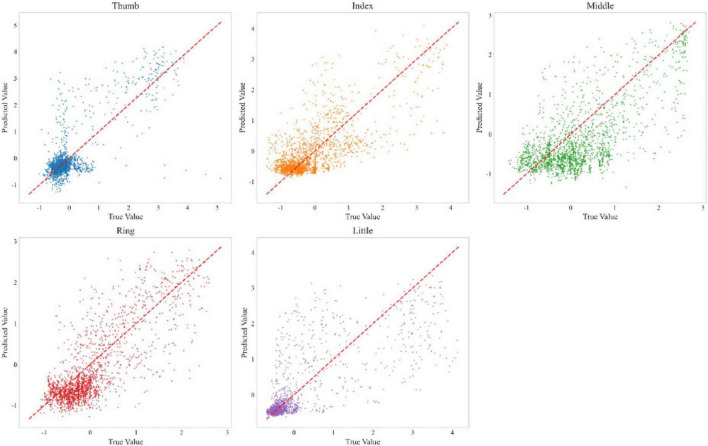
Prediction accuracy scatter plot matrix for individual finger movements.

To ensure the statistical significance of our results and address the inherent non-determinism of neural network training, we conducted multiple independent training runs with different random seeds. Figure 6 shows a performance comparison between our method and the FingerFlex baseline method. The results show that our proposed method achieves significant performance improvements, which are not due to random variations. In addition, Figure 7 shows the correlation between the predicted values and actual values for each finger in the form of a scatter plot. Points closer to the diagonal line indicate higher decoding accuracy.

### 3.4 Ablation study

Ablation study demonstrates the effectiveness of each component in our proposed method. Our full model, incorporating Morlet wavelets, dilated convolution, and transposed convolution, achieves the highest average Pearson correlation coefficient of 0.69 across all subjects. The results in Table 3 show the model performance calculated for different variables.

**TABLE 3:** Comprehensive ablation study of wavelet selection and dilated convolution (Pearson correlation coefficient).

Variant	S1	S2	S3	Average
Full Model (Morlet + Dilated Conv + Transposed Conv)	0.71	0.59	0.77	0.69
Haar wavelets	0.68	0.55	0.74	0.66
Daubechies wavelets	0.67	0.52	0.72	0.64
Symlet wavelets	0.69	0.57	0.76	0.67
Morlet + No Dilated Conv + No Transposed Conv	0.53	0.48	0.59	0.53
Morlet + Dilated Conv + No Transposed Conv	0.61	0.50	0.66	0.59
Morlet + No Dilated Conv + Transposed Conv	0.63	0.51	0.68	0.61

Among different wavelet types, Morlet wavelets consistently outperform alternatives. Compared to other wavelets, Morlet wavelets show superior performance with Symlet wavelets achieving the second-best results (average 0.67), followed by Haar wavelets (0.66) and Daubechies wavelets (0.64). This validates our choice of Morlet wavelets for ECoG signal analysis.

The ablation experiments reveal that each architectural component contributes significantly to the overall performance. Removing all enhancements (Morlet + No Dilated Conv + No Transposed Conv) results in the lowest performance (0.53). The combination of all components yields the optimal performance, demonstrating the synergistic effect of our architectural design choices.

## 4 Discussion

This study employs a variety of innovative techniques in finger bending decoding to enhance model accuracy. These techniques include the use of the Morlet wavelet transform for constructing spectrograms, overlapping sliding windows for time series segmentation, dilated convolution for enhanced feature extraction, and transposed convolution for optimizing the reconstruction process. The synergy of these methods allows the model to better capture the complex dynamic changes in time series data.

First, in constructing the spectrogram, this study opts for the Morlet wavelet transform rather than Haar, Daubechies, or Symlet wavelets. While these alternative wavelets offer high computational efficiency, they are less effective in time-frequency localization, and thus cannot capture high-frequency information or non-stationarity in the signal effectively. In contrast, the Morlet wavelet offers excellent time-frequency localization and can accurately extract high-frequency information, making it more suitable for finger bending tasks that exhibit complex dynamic characteristics. We present a comparison of model performance obtained using different wavelets in Table 3.

**FIGURE 8:**
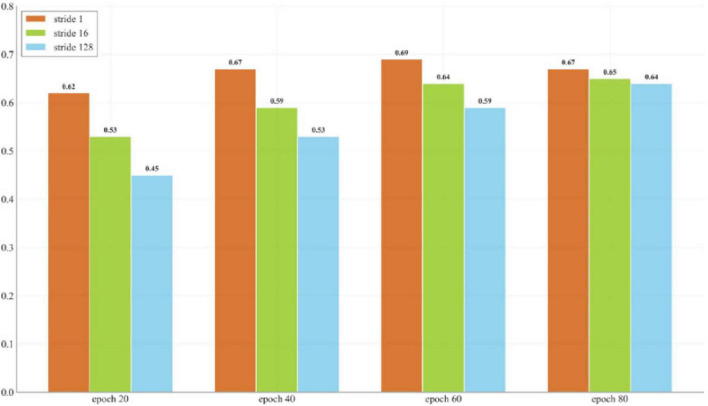
Impact of different window step sizes on model performance and training duration based on the average across all subjects.

For time series processing, this study utilizes overlapping sliding windows to construct a new set of data samples. This approach enables the model to capture finer time variations by dividing a long time series into smaller segments. By using overlapping windows, the original time series generates a greater number of training samples, thereby increasing the available training data. The boundary of each window slides by one step, allowing multiple samples to be derived from the same time series, significantly boosting data diversity. Additionally, overlapping windows retain information from both preceding and succeeding time points, which is particularly beneficial in cases where finger movements transition from a stable state to a bending state. The overlapping time data helps the model understand the previous and subsequent states, thereby improving its ability to decode finger bending. This strategy ensures that adjacent samples exhibit high similarity, which helps the model better capture local changes and long-term dependencies, thereby improving the decoding accuracy. Figure 8 shows the performance test of different window stride sizes and their corresponding epochs. It is worth noting that the performance gap between different stride lengths is more significant in the early training stages, but gradually narrows as training time increases. This indicates that although larger stride lengths can ultimately achieve reasonable performance by extending the training time, stride length = 1 has obvious advantages in terms of data efficiency and convergence speed. Furthermore, a smaller stride is used to minimize temporal differences between samples and mitigate the negative impact of sparse training data on model performance.

In the feature extraction phase, 1D dilated convolution plays a critical role. Since the spatial arrangement of electrodes was unavailable, our method treats each electrode channel as an independent feature dimension rather than relying on spatial topography. The feature reduction layer adapts to this constraint by learning optimal feature representations from the electrode-frequency matrix without assuming spatial relationships. Our method’s performance gains stem from superior temporal modeling rather than spatial priors. The 1D dilated convolution in the encoder enhances interactions across channels and frequency bands and effectively captures the temporal dependencies of signals from different electrodes and frequency ranges. Signals from different electrodes and frequency bands often exhibit unique temporal characteristics, and 1D dilated convolution is well-suited to capture these temporal dependencies without the need to model each electrode and frequency band separately. This operation helps extract deep cross-temporal correlations between different frequencies and channels, which aids in modeling complex signal interactions. Compared to traditional 2D convolution, dilated convolution reduces the model’s input feature dimensions by merging the electrode and frequency dimensions into a single feature dimension, thus improving computational efficiency and avoiding the complexities associated with high-dimensional inputs. Furthermore, compared to standard 1D convolution, 1D dilated convolution offers significant advantages: it achieves a larger receptive field without increasing the number of parameters, enabling more efficient capture of long-range temporal dependencies; and after the feature reduction layer, all electrode channels and fre-quency features are compressed into a one-dimensional vector, and 1D dilated convolution can effectively learn patterns across the temporal dimension from this com-pressed representation, capturing complex temporal interactions that would be difficult for standard 1D convolution to detect within the same parameter budget.

**FIGURE 9:**
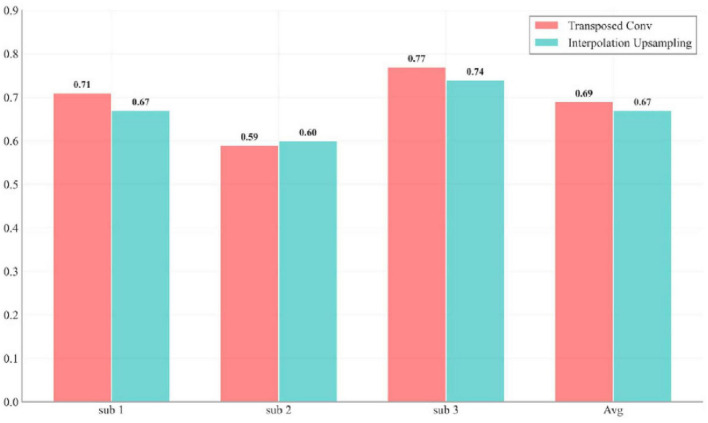
Impact of transposed convolution *versus* linear interpolation upsampling on model performance for each subject.

In the decoding phase, transposed convolution is used to restore temporal resolution and integrate low- and high-level features through skip connections. Unlike the first convolution module in the Decoder, which performs standard convolution, transposed convolution reconstructs detailed information by expanding the size of the input feature map, thereby ensuring the restoration of temporal resolution. Figure 9 visualizes the performance difference between transposed convolution and ordinary linear upsampling. Compared to traditional interpolation-based upsampling methods, transposed convolution offers superior performance in restoring high-frequency details and improving numerical accuracy. While interpolation upsampling is computationally cheaper, it generates new data solely based on existing feature maps, which can result in overly smooth predictions and fail to capture complex dynamic changes such as finger bending. The primary advantage of transposed convolution is its ability to generate appropriate interpolation methods by learning from low-resolution feature maps, not only restoring the temporal resolution but also recovering lost details during downsampling, leading to more refined and coherent predictions.

Higher correlation between test samples and actual results indicates better decoding performance, which means higher decoding accuracy. This means that our algorithm can better assess the extent to which patients with spinal cord injuries or amputations can control prosthetic devices, including basic activities of daily living such as grasping objects and typing.

Although this study makes significant advances in finger bending decoding, there remain some challenges in achieving fine-grained control of motion direction. Specifically, while the model can successfully decode finger movements, there is still a gap between the predicted and actual values, and the model’s output does not perfectly match the true motion trajectory. This small sample size may limit the generalizability of our findings across diverse populations with varying neuroanatomical characteristics, age groups, and motor abilities. Access to larger, multi-center datasets with diverse subject populations would enable more robust validation of our method’s generalizability and facilitate the development of universal decoding models. The computational overhead of our approach, including Morlet wavelet transforms and skip connections architecture, may present challenges for real-time implementation in resource-constrained environments. Future research can focus on improving decoding accuracy and real-time performance, particularly for fine-grained control of motion direction. Additionally, integrating more efficient timing modeling techniques and adaptive algorithms will enhance the system’s adaptability and robustness across various environment.

## 5 Conclusion

In this study, we developed a novel deep learning method to decode the degree of finger bending based on 3D ECoG signals. By constructing 3D data samples and employing an encoder with dilated convolution and a decoder with transposed convolution, our model achieves a significant breakthrough, surpassing an 80% correlation coefficient for single-finger decoding in the BCI Competition IV dataset 4. Compared to previous studies, our model demonstrates an overall performance improvement of 2.98%, with an average correlation coefficient of 0.69 for all fingers across all subjects. Our proposed method holds promise for advancing limb movement control systems based on ECoG signals and highlights the potential of techniques that decode human intentions to enable movement control.

## Data Availability

The original contributions presented in the study are included in the article/supplementary material, further inquiries can be directed to the corresponding authors.
